# Evaluation of the outpatient therapeutic program for severe acute malnourished children aged 6–59 months implementation in Dehana District, Northern Ethiopia: a mixed-methods evaluation

**DOI:** 10.1186/s12887-022-03417-9

**Published:** 2022-06-28

**Authors:** Getachew Teshale, Ayal Debie, Endalkachew Dellie, Tsegaye Gebremedhin

**Affiliations:** Department of Health Systems and Policy, Institute of Public Health, College of Medicine and Health Sciences, University of Gondar, P.O. BOX196, Gondar, Ethiopia

**Keywords:** Evaluation, Outpatient therapeutic program, Implementation, Dehana, Ethiopia

## Abstract

**Background:**

In Ethiopia, about 57% of child mortality is associated with acute malnutrition in which the burden is dominant at the rural community. In that regard, the Ethiopian government has been implementing the Outpatient Therapeutic Program (OTP) for managing the uncomplicated sever acute malnutrition among children aged 6 to 59 months at community level by health extension workers. But nothing is known about the implementation status of OTP. Thus, this evaluation aims to evaluate the implementation status of OTP in Dehana district, northern Ethiopia.

**Methods:**

A facility-based cross-sectional evaluation with concurrent mixed-method was employed from 1st February to 30th April 2020. A total of 39 indicators were used to evaluate the availability, compliance and acceptability dimensions of the program implementation. A total of 422 mothers/caregivers for exit interview, 384 children’s (diagnosed with acute malnutrition) record reviews, nine key informants’ interview, and 63 observations were done in this evaluation. A multi-variable logistic regression analysis was used to identify the predictor variables associated with acceptability. Adjusted Odds Ratio (AOR) with 95% confidence interval (CI), and *p*-value < 0.05 were used to declare statistically significant variables. The qualitative data were tape recorded, transcribed in Amharic and translated into English and finally thematic analysis was done.

**Results:**

The overall implementation of OTP was 78% measured by availability (87.5%), compliance (75.3%), and acceptability (71.0%) dimensions. Trained healthcare providers, Ready to Use Therapeutic Food (RUTF), Mebendazole, and Oral Rehydration Salt (ORS) were available in all health posts, whereas vitamin A and folic acid were stocked out in some health posts. The health care providers complained that interruption of supplies, work overload and improper usage of RUTF by caregivers were the common challenges of program delivery. Rural residence (AOR = 0.18, 95% CI: 0.09–0.39), knowledge on childhood malnutrition and program services (AOR = 2.27, 95% CI: 1.04–4.97), and had malnourished children previously (AOR = 1.82, 95% CI: 1.01–3.30) were significantly associated with the acceptability of OTP program.

**Conclusion:**

The overall implementation status of OTP was judged fair. Low achievement was observed on the compliance of health care providers to the standards, and acceptability of program services. Therefore, the program needs great improvement to enhance the outcome of childhood malnutrition management.

## Background

Acute malnutrition is a nutritional deficiency resulting from either inadequate energy or protein intake. Children with acute malnutrition are common in developing countries as a result of inadequate food supply caused by social, economic, and environmental factors [1]. Severe acute malnutrition (SAM) among children aged less than five years is defined as very low weight for height (below -3z scores of the median World Health Organization (WHO) growth standards) or (below 70% of the median of national center for health statistics standard) and/or the presence of bilateral pitting edema [2]. In children aged 6–59 months, a middle-upper arm circumference (MUAC) less than 11.5 cm is also indicative of SAM [3].

Childhood malnutrition is one of the major global health problems, contributing to childhood morbidity, mortality, impaired intellectual development, suboptimal adult work capacity, and increased risk of diseases in adulthood [4]. Globally, nearly 60 million children suffered from SAM. Moreover, SAM is one of the commonest reasons for pediatric hospital admission and 25–30% of child deaths was related with SAM during hospital admission in many low income countries [5].

Ethiopia is one of the countries with the highest under-five children mortality rate and malnutrition contributed 57% of all childhood deaths [6]. According to the 2016 Ethiopian Demographic Health Survey (EDHS) report children who were stunted (38%), wasted (10%) and underweight (24%) [7]. Even though the incidence of acute malnutrition has shown a decrement in Ethiopia, it is still the major contributors to under-five children morbidity and mortality in Waghmera zone, including Dehana district (Dehana District Health Office: Annual district health reports, unpublished). SAM has been managed by admitting cases in the inpatient facilities. Treatment is matched to the nutritional and clinical needs of the child, with the majority of children receiving treatment at home using ready-to-use foods. However, this traditional therapeutic feeding center (TFC) model of inpatient care was unable to provide an effective response to a large-scale humanitarian crisis as poor access due to considerable obstacles leading to have limited coverage [8].

Community-Based Management of Acute Malnutrition (CMAM) is a decentralized community-based approach to treating acute malnutrition. It consists of four components: (1) stabilization care for acute malnutrition with complications, (2) out-patient therapeutic care for severe acute malnutrition without complications, (3) supplementary feeding for moderate acute malnutrition and (4) community mobilization [9]. In-patient care is provided only for complicated cases of acute malnutrition. Admission of SAM without complication in the stabilization center may result in the risk of hospital-acquired infection and caregivers (usually mothers) spend their time away from their family. This condition can also results in malnutrition among other children waiting at home that leads to increase the defaulter rate [10]. Outpatient therapeutic program (OTP) is established to provide home-based treatment and rehabilitation for children 6–59 months old with SAM without medical complication/s and good appetite to reduce these challenges [2].

**Fig. 1:**
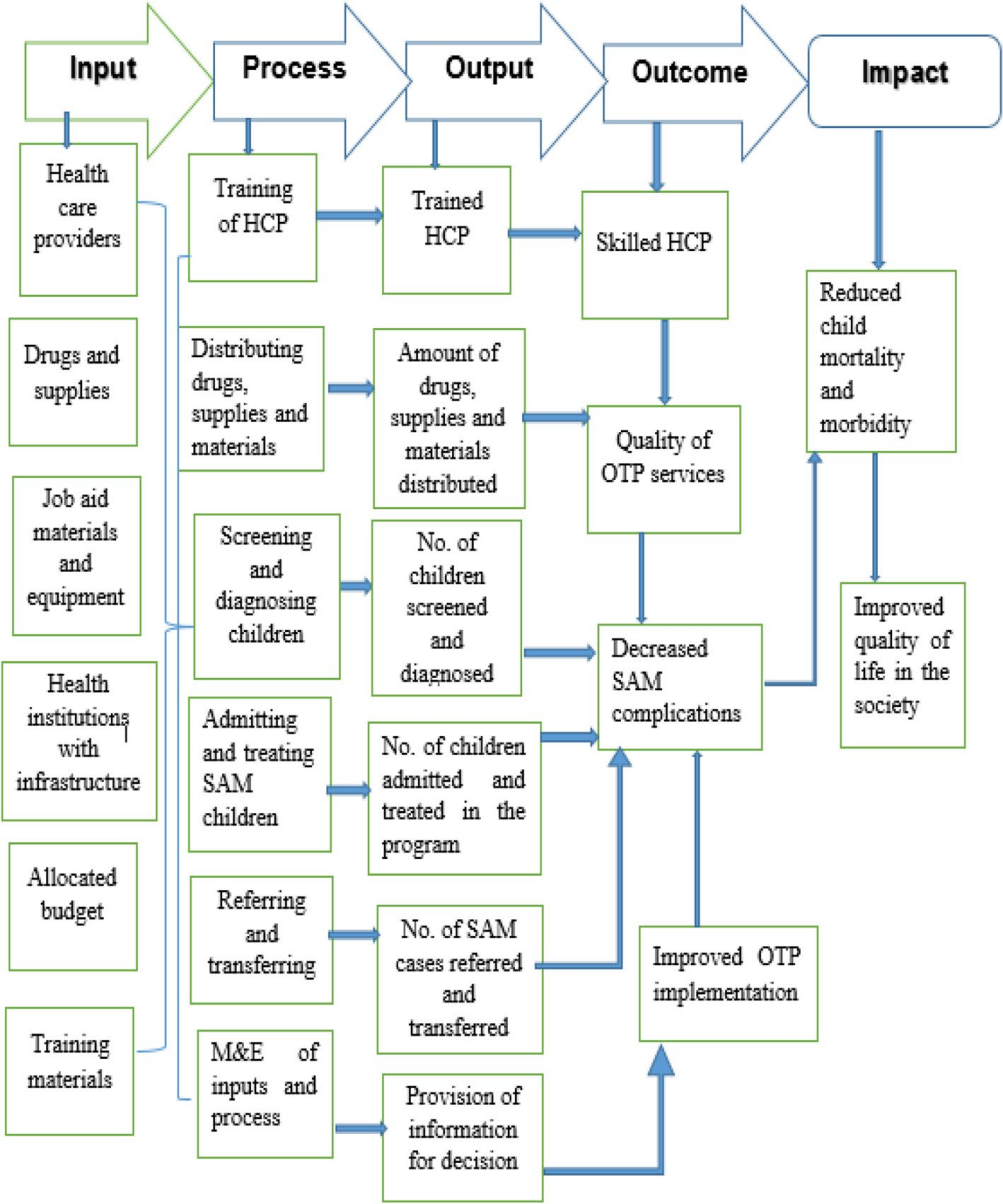
Logic model of OTP Program in Ethiopia

SAM is a major global public health problem that affects over 20 million children and contributed 1–2 million child mortality [5]. The OTP logic model was presented in Fig. 1. Accordingly, the number of children screened for malnutrition, diagnosed, admitted and tread based on the protocol in the program are the expected output of the program activities. The number of children recovered, died, defaulted, transferred, and referred among those who are admitted to the program are considered as the outcome of the program. Reduction of SAM incidence and prevalence, the child mortality rate due to SAM, and improving quality of life in the society are the distant outcomes or impacts of the program.

A study conducted in Uganda indicated the overall program performance of the study sites scored as poor (55%), fair (41.7%), good (3.3%), and excellent (0%). The cure, death, defaulter, and transfer rates were also 52.9, 0.1, 38.2, and 4%, respectively [11]. An outcome evaluation conducted in Wolaita zone shows that the recovery rate, death rate, average weight gain, and average length of stay were 64.9, 1.2, 4.2 g/kg/day, and 3.2 weeks, respectively, the results were below the sphere standards except the average length of stay [1]. Another study in the Tigray region showed the recovery rate (61.7%), death rate (3.02%), and the average length of stay (6.24%) [6].

The high mortality rate in children aged under 5 years in relation with SAM is due to poor case management. Well implemented OTP reduces case fatality rates due to malnutrition. Even though the program is matured to be evaluated at the various levels of its implementation (process, outcome or impact), more of the studies at the country level focused on the outcome and impact of the program. The evaluation was followed participatory approach through engaging the potential stakeholders for the program. The identified potential stakeholders were presented in Table 1. Thus, this can help as an input for new nutritional service program development and existing program improvement that aimed to evaluate the implementation of OTP for management of SAM and identify the challenges at the input, process (activities) and output level of the program for children aged 6–59 months in Dehana district, northern Ethiopia.

**Table 1:** Stakeholder analysis for OTP evaluation in Dehana District, Northern Ethiopia, 2020

S. No	Stakeholders	Role in the program	Role in the evaluation	Perspectives in the evaluation	Means of communication
1	Health care providers	Program Implementers	Information sources, involve during evaluation planning	Evaluation result users	Interview, observation Sessions/ discussion
2	District health office	Program supporters and Implementers	Information sources, involve during evaluation planning	Evaluation result users for decision making on OTP improvement	Formal letters Key informant interview Document review Discussion
3	NGO (plan international Ethiopia)	Funder, support supplies, provide training	Information sources involve during evaluation planning	Use evaluation result to identify the support needed areas	Key informant interview
4	Children 6–59 months’ caregivers	Program consumers/beneficiaries/	Information sources	Evaluation result beneficiaries through improved service delivery	interviewer-administered interview
5	Zonal health district,	Funders, supply and training provider	Information sources	Finding users	Through reading guidelines, policies,
6	Regional health bureau,	Funding, supply and training provider	Evaluation fund provider	Finding users	A formal letter, through reading guidelines, policies,

## Methods and materials

### Evaluation settings and period

The evaluation was conducted in Dehana district, Waghmera zone in northern Ethiopia. Dehana district is one of the six districts of Waghimra administrative zone in Amhara National Regional State, Ethiopia and bounded by Ziquala in the North, Sekota in the East, Gazgibla and Bugna in the South, and Ebnat district in the West. It has 1 urban and 27 rural kebeles (lowest administrative unit in Ethiopia). According to the 2014/2015 population and housing census estimation of Ethiopia, the population of Dehana district were 109,687 of which 55,062 were females and about 94.5% of the district’s population lived in the rural area [12]. The most common topographic feature of the district is characterized by a chain of mountains and deeply incised valleys. The community uses mixed farming (crop production and livestock rearing) [13]. The farming activity is highly affected by low annual rainfall and difficulty of topography. The district has 31 health posts, 6 health centers, and one primary hospital (Dehana Woreda health office: Woreda Health sector report, unpublished). Evaluability assessment was done from 01 to 15 December 2019 as a pre-evaluation activity and the actual evaluation was conducted from the 1^st^ of February to 30^th^ of April 2020.

### Evaluation design

A cross sectional with concurrent mixed methods evaluation was done. Both qualitative and quantitative data were collected concurrently and integrated at the interpretation phase. The qualitative data were used to complement the quantitative findings.

### Evaluation approach and dimensions

A formative evaluation approach that focused on the process theory of the OTP was used to evaluate the implementation. The availability and acceptability dimensions from the access framework [14] and compliance dimension from the fidelity framework [15] were used to evaluate the implementation of OTP.

### Population and samplings

All children aged 6 to 59 months diagnosed with SAM and enrolled in OTP program and their mothers/ caregivers, program implementers (health extension workers, health care providers, health care managers, and other stakeholders), OTP records and registries in the health posts of Dehana district were included in the evaluation.

The sample size was determined by the three dimensions. Accordingly, Since there is no previous study on compliance of HEWS in our study area, sample size for measuring the compliance of the program was calculated using a single population proportion formula by considering 50% of health extension workers (HEWs) are complied with the standard guidelines, 95% confidence level (CI) and margin of error (d) 5% which yields 384. Thus, a total of 384 children’s charts that had been managed under OTP were reviewed for compliance of HEWs to the program protocol. The sample size to measure the mothers’/caregivers’ acceptability towards OTP was calculated by considering a 50% of clients accepted the program, 95% CI, 5% of margin of error and 10% non- responses, which gave 422. To assess the availability of required supplies, materials and human resources for the program, nine randomly selected health posts (30% of the total health posts in the district according to the WHO recommendation for the assessment of operationality of district health system) [16] were included. A total of 63 non-participatory patient-provider interaction were observed. Eleven key informants (9 HEWs, district health office nutrition focal, and plan international officer) were interviewed. First, nine health posts were randomly selected using lottery method. In these health posts, OTP records and documents were reviewed. A sample to population (children diagnosed and enrolled in OTP per health posts) proportion of mothers/caregivers and documents were done in each health post. The registration was assessed starting from the last registration number until the required sample size was obtained in each selected health posts. All caregivers attending the OTP sites during data collection were selected consecutively and interviewed towards the program acceptability. However, those seriously ill caregivers/ mothers were excluded from interviews.

### Measurements of variables

Uncomplicated SAM among children aged 6–59 months is the weight-for-height of children below 70% or below -3z score of the WHO standards and/or bilateral pitting edema or MUAC < 11.5 cm [2].

Availability of resources including trained health care providers were measured using 14 indicators and we gave a weighted value of 40.

Compliance was used to measure the adherence of HEWs to the OTP treatment guidelines through observation of patient-provider interaction and document reviews. To measure this, we used 11 indicators and a weighted value of 30 was given.

Acceptability of OTP service by mothers/ caregivers was measured using 14 indicators and had a weighted value of 30. The indicators were developed with a five-point Likert scale (1: strongly unacceptable, 2: unacceptable, 3: neutral, 4: acceptable and 5: strongly acceptable). Those mothers/caregivers who scored above 60% of the total acceptability measuring score were considered as “acceptable”, otherwise “not acceptable”. The cutoff point for this categorization was calculated using the demarcation threshold formula ($$\mathrm{cutoff point}=\frac{(overall highest score-overall lowest score}{2 }+total lowest score$$). The independent variables for the acceptability dimension were socio-demographic and economic variables (age, educational status, religion, ethnicity, economic status, occupational status, parity), knowledge of mothers/caregivers, availability of drugs, and visiting time by HEWs.

These indicators were developed from the national SAM implementation guideline and other related evaluations using nominal group technique participation of stakeholders. Weight was given for each of the selected indicator by the stakeholders before the evaluation had been initiated. Indicators score was calculated using the formula ($\mathrm{Indicator weight}=\frac{observed number X Indicator weight}{expected number}$).

The overall implementation of OTP was the outcome variable of the evaluation as measured by 39 indicators over the three dimensions: availability (14 indicators), compliance (11 indicators) and acceptability (14 indicators). Finally, the implementation status of the program was categorized and judged as; poor, fair and good if the scores were < 60%, 60–80% and ≥ 80%, respectively.

### Data collection tools and procedures

Resource inventory and data extraction checklists were adapted from the evaluation of community management of acute malnutrition (CMAM) Ethiopia [9]. A structured interviewer administered questionnaire was adapted from reviewing of related literatures to measure the acceptability of OTP. The key informant interview (KII) guide was also developed to explore the views of KIs. The interview questionnaire and guide were initially developed in English and translated into the local language (Amharic) and finally back to English to ensure consistency. All the interviews were done in Amharic. Four trained clinical nurses and two health officers were recruited from Sekota and Gazgebla health centers as data collectors and supervisors, respectively. Before data collection, two days training was given on the overall objective of the evaluation and basic techniques of data collection procedures. Then both quantitative and qualitative data were collected parallelly. The quantitative data were collected from program documents and charts of the admitted children in OTP program. Qualitative data were collected through interviewing the district nutrition focal person, health care providers, plan international organization Dehana district officers, caretakers of children and non-participatory observations of patient-provider interaction. Key informant interviews were done to answer why and how questions to explore the gaps and best experiences of the OTP program implementation.

Pretest was done among 20 mothers/caregivers to identify the misunderstood items and necessary corrections were made based on the pretest findings. Pre-test of the data extraction and resource inventory checklists were done at Menshewa health post (non-selected health post) to check the fitness of checklists with the registration format and the required resources for the program. The data collectors were supervised, and the completeness and consistency of data were checked and managed by the principal evaluator daily. To assure the qualitative data quality, an experienced qualitative data collector (principal evaluator) was involved and finally, voice insertion was done in the findings to increase its credibility.

### Data management and analysis

Quantitative data were entered to Epi-data and exported to Statistical Package for Social Sciences (SPSS) version 25 software for analysis. Descriptive statistics used were presented in narrations and tabular forms. The overall implementation of the program was analyzed and interpreted as a combination of the availability, compliance and acceptability dimensions based on predetermined judgment parameters. A binary logistic regression analysis was done to identify the factors associated with OTP acceptability. Variables with a *p*-value of less than 0.2 during bi-variable analysis were taken into multi-variable analysis. Finally, those variables with a *p*-value of less than 0.05 and Adjusted Odds Ratio (AOR) with 95% Confidence Interval (CI) were declared as statistically significant variables. Qualitative data were tape-recorded, transcribed into Amharic and translated to English language. The translated data were coded and analysed thematically.

### Judgment matrix analysis

The judgment matrix was adapted from sphere standards with the involvement of stakeholders. The standardized indicators and categories for better CMAM reporting in 2015 [17]. The weighted values for availability, compliance and acceptability dimensions were given based on the stakeholders and investigator agreement on each indicator. Based on the agreement, availability dimension had a weighted value of 40, compliance dimension had 30 and acceptability dimension had 30. The judgment parameter for availability, compliance and acceptability dimensions and the overall implementation of the program were categorized as poor (< 60%), fair (60–80%) and good (≥ 80%).

## Results

In this evaluation, a total of nine health posts inventory, 11 key informants’ in-depth interview, 63 client and provider’s interaction observation, 422 mothers/caregivers exit interview, 384 children’s chart review, and 63 months report review were done.

### Availability of resources

A total of 21 HEWs were assigned over the nine health posts; an average of more than two HEWs per health post. Of these HEWs, 95.2% of them were trained on SAM management. Most HEWs who participated in KIIs also confirmed that training in OTP had been given frequently. Even if trained HEWs were assigned in each health post 77.7% of them complained that they could not cover the workload at the health posts. The key informant interview responses also supported that shortage of HEWs at the health posts were the problem to implement the program properly.

“We received OTP training in the recent training session delivered on the new OTP admission and discharge criteria. Besides, the woreda health office’s health professionals and plan international Ethiopia provide on job trainings and technical supports for us.” (31 years old HEW).

“There are many activities other than the OTP services in the health posts, especially the health insurance registration, renewing, and outreach activities are very tiresome. All the activities may not be covered with the available health workforce and it needs additional HEWs.” (25 years old HEW).

Some medicines, such as RUTF, drugs for deworming, and ORS were 100% available in all the health posts. Antibiotics, vitamin A and folic acid were also 88.8%, 77.8%, and 88.9% available in the health posts, respectively. About 22% and 11% of health posts did also not fill Intra-facility Re-supply Request form (IRRF) and bin card for vitamin A and folic acid, respectively in the last six months. HEWs complained that OTP supplies were not available as per the required quantity. The key informants were explained these findings as:

“Most of the time, the inputs for OTP are available in the woreda health office but we did not get within the required time and amount at health posts. As a result, we are trying to solve this problem through sharing the resources from other health posts and/or the nearby health centers.” (26 years old HEW).

“The problem is not only resulted from the shortage of the supplies but also the absence of transportation access, especially, in the summer seasons the roads were destructed by floods. Moreover, the HEWs did not filled the request form as per the schedule before the supplies are running out.” (Dehana District Nutrition focal person).

Only one-third (33%) of the health posts had a clean water source for drinking and washing of equipment but none of them did not have soap for handwashing. Almost all available drugs at the health posts had an expired date of three or more months except amoxicillin which would expire within a month. All health posts stored their available resources in a dry and clean area. The key informant affirmed that;

“Only few health posts had a clean water source. These health posts had water tankers and these tankers were constructed by Plan International, Ethiopia. Moreover, avoiding of wastage of drugs and other supplies also the main activities of non-governmental organizations (NGOs) and the local health sector governmental organizations. Constructing water tankers and painting the walls of the health posts would be encouraged and supported by Plan International, Ethiopia.” (35 years old Plan International officer, Ethiopia).

All observed health posts had SAM protocols, OTP quick references, MUAC classification table, MUAC tape, electronic scale, RUTF ration card, and OTP card. Most of the health posts had SAM classification algorithm (89%), Integrated Management of Newborn and Childhood illness (IMNCI) protocol (89%), and monthly report forms (77.8%). Only few health posts had length board (11%), standing meter (44%), and referral forms (44%) (Table 2). The HEWs complained that the absences of electric power and printing machines in the health posts or their cluster health centers were the challenges to use and make ready referral and report forms.

“We have no referral and reporting formats. We referred cases to health centers or hospital with white paper. We also used white papers for reporting of activities. This might be due to the absence of light and printing machines in the health posts and/or in the cluster health centers.” (30 years HEW).

**Table 2:** Availability of medicines and supplies for OTP in Dehana District, Northern Ethiopia, 2020

Drugs and supplies	Available at time of observation, *n* = 9 (%)	Available in the last 6 months, *n* = 9 (%)
RUTF	9(100.0)	9(100.0)
Vitamin A	7(77.8)	7(77.8)
Amoxicillin capsule	9(100.0)	8(88.8)
Amoxicillin syrup	8(88.8)	8(88.8)
Folic acid	8(88.8)	8(88.8)
Mebendazole /Albendazole	9(100.0)	9(100.0)
ORS	9(100.0)	9(100.0)
Soap for washing	0 (0.0)	0 (0.0)
Water for drinking	3 (33.3)	3(33.3)
SAM protocol	9 (100.0)	9 (100.0)
OTP quick reference	9 (100.0)	9 (100.0)
SAM classification algorithm	8 (88.8)	8 (88.8)
MUAC classification table	9 (100.0)	9 (100.0)
IMNCI protocol	9 (88.8)	9 (88.8)
MUAC tape	9 (100.0)	9 (100.0)
Electronic scale	9 (100.0)	9 (100.0)
Length board	1 (11.0)	1 (11.0)
Standing meter	4 (44.4)	4 (44.4)

The overall availability of resources for OTP implementation was 87.5% which was good based on our presetting judgment parameter. Percentage of OTP sites having anthropometric measuring equipment were judged as fair. Moreover, percentage of OTP sites having weight for height reference card and referral form availability were poor in the areas (Table 3).

**Table 3:** Availability resources performance indicators in the implementation of OTP in Dehana District, Northern Ethiopia, 2020, *n* = 9

Availability indicators	E^*a*^	O^*a*^	W^*a*^	S^*a*^	A^*a*^	JA^*a*^
Proportion of trained healthcare providers	18	18	10.0	10.0	100.0	Good
% of OTP sites having RUTF	9	9	3.0	3.0	100.0	Good
% of OTP sites having antibiotics	9	9	3.0	3.0	100.0	Good
% of OTP site having non-antibiotic OTP medications	9	8	4.0	3.6	88.9	Good
% of OTP sites having SAM protocol	9	9	2.0	2.0	100.0	Good
% of OTP sites having OTP quick reference	9	9	2.0	2.0	100.0	Good
% of OTP sites having SAM classification algorism	9	8	2.0	1.8	90.0	Good
% of OTP sites having IMNCI protocol	9	8	1.0	0.9	90.0	Good
% of OTP sites having anthropometric measuring equipment	9	6	6.0	4.0	66.7	Fair
% of OTP sites having Weight for height reference card	9	4	2.0	0.9	45.0	Poor
% of OTP sites having OTP card	9	9	1.0	1.0	100.0	Good
% of OTP sites having referral form	9	4	1.5	0.7	44.4	Poor
% of OTP sites having RUTF ration reference card	9	9	1.0	1.0	100	Good
% of OTP sites having OTP Report form	9	7	1.5	1.2	80.0	Good
Over all availability of the program	9	8	40	35	87.5	Good

^*a*^*E* Expected, *O* Observed, *W* Weight, *S* Score = (Observed X weight)/Expected), *A* Achievement in percentage = (S/W) * 100), *JP* Judgement Parameter

### Compliance of HEWs to OTP standards and guidelines

From the 63 patient-provider interactions, we found that only 46.6%, 95.2%, and 14.3% of the children were checked for bilateral pitting edema, weight and height, respectively. Nearly ninety-eight and ninety percent of the children’s MUAC was measured and medical complications were assessed, correspondingly. Moreover, 92% of malnourished children were admitted based on the admission criteria and appetite test was done for 47.6% of the children during the admission and follow-up time.

From the total of 384 children’s registrations reviewed, medical history and physical examination was done for 51.6% children and RUTF was calculated and registered for 88.8% of the children among those who needs it. The registration was completed for only 32.2% of the children and 97.7% of admitted children were discharged from the OTP program based upon the discharge criteria (Table 4). Based on our observation and OTP document review, 75% of the HEWs were complied with the OTP protocol and judged as fair (Table 5).

**Table 4:** Observation and routine document review on compliance of HCPs in Dehana District, Northern Ethiopia, 2020

**Activities (Observation)**	**Tasks performed by HEWs (***n*** = 63 (%)**
Check bilateral edema	30 (47.6)
Measuring MUAC	62 (98.4)
Measuring weight	60 (95.2)
Measuring height	9 (14.3)
Check for medical complications	57 (90.5)
Observe appetite test	30 (47.6)
Admit based on admission criteria	58 (92.1)
**Checked in the registration book**	**Tasks performed by HEWs (*n* = 384), n (%)**
Children medical history and physical examination recorded	198 (51.6)
Medication given registered	247 (64.3)
RUTF needed calculated and registered	341 (88.8)
Children discharged based on the protocol	375 (97.7)
Children’s registration completed	124 (32.3)
Monthly report prepared and send to woreda health office	63 (100)

**Table 5:** Performance of compliance of OTP implementation in Dehana District, Northern Ethiopia, 2020, *n* = 63

Compliance measuring indicators	E^a^	O^a^	W^a^	S^a^	A^a^	JP^a^
% of children bilateral pitting edema checked during the observation	63	30	2.0	0.9	45.0	Poor
% of children whose anthropometry is measured	63	44	6.0	4.2	70.0	Fair
% of children classified accordingly during the observation	63	54	2.0	1.7	85.0	Good
% of children admitted according to admission criteria during the observation	63	58	2.0	1.8	90.0	Good
% of children whose medical complication is checked during observation	63	57	2.0	1.8	90.0	Good
% of children whose appetite is tested upon admission and follow-up during the observation	63	30	2.0	0.9	45.0	Poor
% of admitted children whose medical History and Physical examination recorded on the OTP chart	384	198	3.0	1.5	50.0	Poor
% of admitted children whose routine medication given recorded on the OTP chart	384	247	3.0	1.9	63.3	Fair
% of admitted children whose amount of RUTF needed is recorded on the OTP chart	384	341	2.0	1.8	90.0	Good
% of children discharged according to the protocol	384	375	3.0	2.9	96.7	Good
% of OTP monthly report prepared and reported to the next level	9	9	3.0	3.0	100.0	Good
Over all compliance			30	22.5	75	Fair

^a^*E* Expected, *O* Observed, *W* weight, *S* Score = (Observed X weight)/Expected), *A* Achievement in percentage = (S/W) * 100), *JP* Judgement Parameter

### Acceptability of OTP services

#### Socio-demographic and obstetric characteristics of participants

Table 6 shows the socio-demographic and obstetric characteristics of the mothers/caregivers who participated for the assessment of OTP acceptability. All mothers/caregivers were Orthodox Christians; 80.3% were married; and 66.8%were uneducated. Nearly seventy two percent were farmers; 82% were living in rural areas. Besides, 52.6% of the admitted children aged 6 to 59 months were males. Almost twenty nine percent of mothers/caregivers had more than three gravidities and 9.4% of mothers/caregivers had history of still births.

**Table 6:** Sociodemographic characteristics of respondents on the evaluation of OTP in Dehana District, Northern Ethiopia, 2020

Variables	Categories	Frequency (*n* = 422)	Percent (%)
	< 30	209	49.5
	> 30–39	212	502
Current maternal marital status	Single	83	19.7
	Married	339	80.3
Maternal education	Uneducated	282	66.8
	Educated	140	33.2
Paternal education	Uneducated	247	58.5
	Educated	175	41.5
Maternal occupation	Unemployed	96	22.7
	Gov't employers	20	4.7
	Farmers	306	72.5
Paternal occupation	un employers	67	15.9
	Gov’t employers	40	9.5
	Farmers	307	72.7
Family size	≤ 4	243	57.6
	> 4	179	42.4
Residence	Urban	76	18.0
	Rural	346	82.0
Average annual HH income (ETB)	< 36,900	239	56.6
	≥ 36,900	182	43.1
Child sex	Male	222	52.6
	Female	200	47.4
Age of child (in months)	6–12	69	16.4
	12–24	183	43.4
	24–36	136	32.2
	> 36	34	8.1

#### Knowledge, adequacy of RUTF and obstetric history of mothers/caregivers

The knowledge and obstetric history of mothers/caregivers were presented in Table 7. Only 25.4% of mothers/caregivers knew about child malnutrition and 17.1% distinguished the signs and symptoms of malnutrition. One-fifth properly explained the cause of childhood malnutrition. Almost half (48.1%) of mothers had run-out of RUTF before the appointment day. On the other hand, 26.3% of mothers (caretakers) of children had an extra RUTF during the next appointment. Thirty percent (28.9%) of mothers had more than three gravidities and 9.4% of them had history of still births. Moreover, 14.2% of mothers/caregivers’ children had history of malnutrition.

**Table 7:** Knowledge and obstetric history of mothers (caretakers) in Dehana District, Northern Ethiopia, 2020

Variables	Categories	Frequency (*n* = 422)	Percent (%)
Knows about child malnutrition	No	315	74.6
	Yes	107	25.4
Distinguished child malnutrition signs and symptoms	No	350	82.9
	Yes	72	17.1
Knows about the cause of child malnutrition	No	343	81.3
	Yes	79	18.7
Knew about OTP program	No	387	91.7
	Yes	35	8.3
Facing run-out of RUTF before next appointment	No	219	51.9
	Yes	203	48.1
Having excess RUTF at the end of appointment	No	311	73.7
	Yes	111	26.3
Gravidity	≤ 3	300	71.1
	> 3	122	28.9
Parity	≤ 3	302	71.6
	> 3	120	28.4
Still births	No	381	90.3
	Yes	40	9.4
Had malnourished children previously	No	362	85.8
	Yes	60	14.2

### Acceptability of OTP services

Table 8 showed as the acceptability of OTP using the measuring items. Accordingly, the overall acceptability of OTP services was 71% which was judged as fair. Low proportion of acceptability was measured on traveling distances, transportation access and fees to receive the services and the difficulty they faced to feed RUTF for their child. On the other hand, high proportion of acceptability was measured on the important of the OTP program for uncomplicated SAM management and the instructions they received from the providers on how to feed the child and when to return for the next appointment. However, the KII at one of the health posts stated the main problem they are facing that;

“Mothers/caregivers took the prescribed RUTF for their child and some of them also sold it in the market as a commodity instead of giving to their malnourished children. Some of them also share with other family members as normal food. This is the main problem of a program on the community or parents’ perspective.” (31 years HEW).

**Table 8:** Performance indicators of acceptability of OTP service by caretakers (mothers) in Dehana District, Northern Ethiopia, 2020, *n* = 422

Acceptability measuring indicators	E^a^	O^a^	W^a^	S^a^	A^a^	JA^a^
Proportion of caretakers whose distances to receive the services are not too far	422	177	2.1	0.9	41.9	Poor
Proportion of caretakers whose access to transportation to the OTP site are fair	422	184	2.1	0.9	43.6	Poor
Proportion of caretakers who said the transportation fees to the OTP sites are fair	422	131	2.2	0.7	31.0	Poor
Proportion of caretakers who replied the schedule or working hours of the OTP sites are appropriate	422	402	2.1	2.0	95.2	Good
Proportion of caretakers who received instructions from the providers on how to feed the child and when to return for the next appointment	422	416	2.0	2.0	98.6	Good
Proportion of caretakers who spent reasonable amount of time to receive the services	422	331	2.2	1.7	78.4	Fair
Proportion of caretakers who received counseling about child nutrition and other things by providers upon their arrival	422	400	2.1	2.0	94.7	Good
Proportion of caretakers who liked discussion about child malnutrition	422	363	2.1	1.8	86.0	Good
Proportion of caretakers who were well informed about child malnutrition by health care provider	422	409	2.1	2.0	96.9	Good
Proportion of caretakers who had friendly approach with healthcare providers	422	407	2.0	1.9	96.5	Good
Proportion of children who did not face difficulty to feed RUTF for their child	422	202	2.3	1.1	47.8	Poor
Proportion of caretakers who did not recommend to share the RUTF with other family members	422	397	2.1	2.0	94.1	Good
Proportion of caretakers who aware selling or buying of RUTF from the market is impossible	422	395	2.2	2.1	93.6	Good
Proportion of caretakers who knew OTP services are important for uncomplicated SAM management	422	420	2.4	2.4	99.6	Good
Overall acceptability of OTP service by caretakers (mothers)			30.0	21.3	71.0	Fair

^a^*E* Expected, *O* Observed, *W* Weight, *S* Score = (Observed X weight)/Expected), *A* Achievement in percentage = (S/W) * 100), *JP* Judgement Parameter

#### Overall implementation of OTP program

The implementation of OTP provision in Dehana district was judged as 78.0% measured by availability (87.5%), compliance (75.3%) and acceptability (71.0%) dimensions (Table 9).

**Table 9:** Overall process evaluation of OTP evaluation in Dehana District, Northern Ethiopia, 2020

Dimensions	E	O	W	S	A (%)	Judgment (G, F or P)^a^
Availability	40	35	40	35	87.5%	Good
Acceptability	30	22	30	22	75.0%	Fair
Compliance	30	21	30	21	71.0%	Fair
Overall implementation of OTP	100	78	100	78	78%	Fair

^a^*G* Good, *F* Fair; *P* Poor

#### Factors associated with acceptability of OTP

In the final multivariable logistic regression analysis, residence, knowledge of caregivers on child malnutrition and mothers who had children with malnutrition previously were statistically significant with acceptability of OTP services as presented in Table 10. The odds of acceptability of OTP services among mothers who lived in a rural area were 0.18 (95% CI: 0.09–0.39) compared with urban residence. The odds of OTP services acceptability among mothers who had malnourished children previously were 1.8 compared the odds of OTP acceptability among their counter parts (AOR = 1.82, 95% CI: 1.01–3.30). The odds of acceptability of OTP services among mothers who knew about child malnutrition were 2.3 compared with the odds of acceptability of OTP among those who did not know (AOR = 2.27, 95% CI: 1.04–4.97).

**Table 10:** Factors associated with acceptability of OTP services in Dehana District, Northern Ethiopia, 2020

Variables		Acceptability		COR (95%CI)	AOR (95%CI)
		Good	Poor		
Current maternal marital status	Single	55	28	1	1
	Married	184	155	0.60(0.37–0.99)	0.88(0.5–1.56)
Maternal education	Uneducated	137	145	1	1
	Educated	102	38	2.8(1.8–4.4)	1.57(0.93–2.65)
Family size	≤ 4	151	28	1	1
	> 4	88	155	0.59(0.39–0.87)	0.75(0.49–1.15)
Residence	Urban	67	145	1	1
	Rural	172	38	0.13 (0.64–0.27)	0.18(0.09–0.39) *
Gravidity	≤ 3	178	84	1	1
	> 3	61	99	0.69(0.45–1.05)	1.00(0.60–1.67)
Knew about OTP program	No	28	187	1	1
	Yes	155	52	4.35(2.19–8.60)	2.27(1.04–4.97) *
Had malnourished children previously	No	162	200	1	1
	Yes	21	39	1.5(0.85–2.66)	1.82(1.01–3.30) *

^*^Significant at *p*-value < 0.05

## Discussion

The implementation status of OTP was judged using three dimensions including availability, compliance and acceptability of the program. In this evaluation, the overall implementation status was judged as poor (< 60%), fair (60–80%) and good (≥ 80%). This cut off points were determined during evaluability assessment (EA) along with the stakeholders. As a result, we found that the overall implementation status of OTP program was 78%, which was fair as per the presetting judgment parameter (JP). Availability resources for the program implementation was 87% which was judged as good. Compliance of HEWs to the treatment guideline was 75% and acceptability of the program services by users was 71%, which were judged as fair as per the JP.

In this evaluation showed that trained health care providers, RUTF, and Mebendazole/ Albendazole were fully available on the other hand antibiotics, vitamin A, and folic acid were avail 88.8%, 77.8% and 88.8%, respectively which are below the expected standard in the evaluation. The findings were incongruent with the study conducted in southern Ethiopia (availability of RUTF (32.9%) and antibiotics (62.9%)) [18]. The possible reason for this variation might be the differences in road and transportation problems [19]. This evaluation also revealed that the health posts had insufficient SAM classification algorithm, IMNCI protocol, monthly report format, length board, standing meter, and referral forms. This may significantly affect the program implementation and service quality. The availability of these supplies in this evaluation were lower than a study conducted in Uganda (100% available) [11]. This could be due to access to electricity for printing forms and preparing guidelines were difficult in our study area.

Compliance of HEWs to the treatment guidelines in this evaluation was 75%. This finding was lower than the study done in Uganda (77.7%) [11]. This might be due to the shortage of some basic materials for the implementation of OTP program which could lead to the HEWs not adhering in turn lowers their performance.

In this evaluation, the HEWs assessment of SAM cases for bilateral pitting edema, weight, height, MUAC, medical complications and appetite test were very poor. This result was slightly lower than an evaluation in Mali and Uganda [20]. This might be because of HEWs’ work load and insufficient measuring materials for SAM assessment. However, availability of antibiotics, RUTF and vitamin A were higher than an evaluation done in Wolaita zone, Ethiopia [1]. The reason for this variation could be due to the program is currently the government and NGOs give priority for child and maternal nutritional programs.

The overall acceptability of the outpatient treatment program was 71% which was less than the presetting judgment parameter (80%). Half (52.2%) of the caregivers of children had faced difficulty to feed RUTF to their children. This result was relatively higher than the findings in southern Ethiopia who faced difficulty (60%) [18]. Nearly 100% of the caregivers accepted RUTF is important for their malnourished children. This result was relatively greater than the study in Bangladesh (91%) [21].

Acceptability of outpatient treatment programs among caregivers who were well aware about child malnutrition and OTP services were higher than compared with their counterparts. This was supported by the findings in Ghana [22]. This may show that knowledge of caregivers of children on the outpatient treatment program of malnutrition services is more important to accept and utilize the services. Caregivers who had previous experience of child malnutrition were more likely accept the program services than those who had not. The possible reason behind this might be previous experience increases the knowledge and awareness of caregivers on child malnutrition and treatment which may contribute to their acceptance and utilization of the program services. Those mothers who lived in a rural area were less likely to accept the program than urban residences. The reason might be due to rural mothers had poor access to mass media about OTP program.

### Limitations of the evaluation

This evaluation focused only on some process evaluation dimensions of access and fidelity frameworks. As a result, judging the program only using these dimensions might not show the full implementation status of the program. This could be the limitation of our evaluation. The other possible limitation of the evaluation was the Hawthorn effect during observation of the patient provider interaction. To minimize this limitation, the first three observations in each health post were dropped to reduce the Hawthorne effect during the non-participatory observations of patient provider interaction.

## Conclusion

This evaluation assessed the implementation status of outpatient therapeutic program mainly focused on availability, compliance, and the acceptability dimensions. The overall implementation status of the program was judged as fair. The availability dimension is compromised by shortage/ stock out of vitamin A and folic acid in some health posts. Besides, insufficient SAM classification algorithm, IMNCI protocol and different formats hinder this dimension. Urban residence, knowledge of caregivers about child malnutrition and mothers who had malnourished children previously were the factors attributed to the acceptability of the program. It is also better to train more HEWs and community volunteers to reduce the work load of HEWs, screen more malnourished children and providing OTP services for them enhance the implementation status of the program. Materials and supplies shall be delivered directly to the health centers or health posts to solve the transportation problem. Collaboration with the police sector and courts is also better to limit the abuse of OTP resources, such as selling of RUTF. HEWs shall document the assessment findings and the services provided using the registration format to identify their gaps, limitations, and better performances.

## Data Availability

Data will be available upon reasonable request from the corresponding author.

## Abbreviations

ARHB: Amhara Regional Health Bureau
CMAM: Community Management of Acute Malnutrition
Dx: Diagnosis
EDHS: Ethiopian Demographic and Health Survey
FGD: Focus Group Discussion
HCP: Health Care Provider
HEWs: Health Extension Workers
IRRF: Intra-facility Resupply Request Form
ITP: Inpatient Treatment Program
KII: Key Informant Interview
Km: Kilometers
MAM: Moderate Acute Malnutrition
MUAC: Mid-Upper Arm Circumference
NGO: Non-Governmental Organization
OTP: Outpatient Therapeutic Program
RUTF: Ready to Use Therapeutic Feeding
SAM: Severe Acute Malnutrition
SC: Stabilization Center
SFP: Supplementary Feeding Program
SNNP: Southern Nation and Nationality
TFC: Therapeutic Feeding Center
UNICEF: United Nation International Children and Education Fund
UoG: University of Gondar
WHO: World Health Organization

## References

[1] Kabalo MY, Seifu CN (2017). Treatment outcomes of severe acute malnutrition in children treated within Outpatient Therapeutic Program (OTP) at Wolaita Zone, Southern Ethiopia: retrospective cross-sectional study. J Health Popul Nutr.

[2] UNICEF (2012). Evaluation of Community Management of Acute Malnutrition (CMAM): Ethiopia Country Case Study.

[3] UNICEF: Evaluation of Integrated Management of Acute Malnutrition (IMAM): Kenya Country Case Study. New York. 2012.

[4] Lenters LM, Wazny K, Webb P, Ahmed T, Bhutta ZA (2013). Treatment of severe and moderate acute malnutrition in low-and middle-income settings: a systematic review, meta-analysis and Delphi process. BMC Public Health.

[5] WHO, UNICEF (2007). Community-based management of severe acute malnutrition: a joint statement by the World Health Organization, the World Food Programme, the United Nations System Standing Committee on Nutrition and the United Nations Children’s Fund. Community-based management of severe acute malnutrition: a joint statement by the World Health Organization, the World Food Programme, the United Nations System Standing Committee on Nutrition and the United Nations Children’s Fund.

[6] Yebyo HG, Kendall C, Nigusse D, Lemma W (2013). Outpatient therapeutic feeding program outcomes and determinants in treatment of severe acute malnutrition in tigray, northern ethiopia: a retrospective cohort study. PLoS ONE.

[7] Central Statistical Agency (CSA) [Ethiopia] and ICF. Ethiopia Demographic and Health Survey 2016. Addis Ababa and Rockville: CSA and ICF; 2016.

[8] Teferi E, Lera M, Sita S, Bogale Z, Datiko DG, Yassin MA. Treatment outcome of children with severe acute malnutrition admitted to therapeutic feeding centers in Southern Region of Ethiopia. Ethiop J Health Dev. 2010;24(3).

[9] World Vision International. CommunityBased Management of Acute Malnutrition (CMAM). 2017. Available at: https://www.wvi.org/sites/default/files/Community_Based_Management_of_Acute_Malnutrition_Project_Model%20%281%29.pdf.

[10] Collins S, Sadler K, Dent N, Khara T, Guerrero S, Myatt M, Saboya M, Walsh A (2006). Key Issues in the Success of Community-Based Management of Severe Malnutrition. Food Nutr Bull.

[11] Wanzira H, Muyinda R, Lochoro P, Putoto G, Segafredo G, Wamani H, Lazzerini M (2018). Quality of care for children with acute malnutrition at health center level in Uganda: a cross sectional study in West Nile region during the refugee crisis. BMC Health Serv Res.

[12] Central Statistical Agency. 2007 population and housing census of Ethiopia. Addis Ababa: Central Statistical Agency; 2007.

[13] Tamene, Moges. Livelihood Strategies and Food Security of Landless Households in Dehana Woreda of The Amhara National Regional State. 2009. Available at: http://etd.aau.edu.et/handle/123456789/12380.

[14] Obrist B, Iteba N, Lengeler C, Makemba A, Mshana C, Nathan R, Alba S, Dillip A, Hetzel MW, Mayumana I (2007). Access to health care in contexts of livelihood insecurity: a framework for analysis and action. PLoS Med.

[15] Carroll C, Patterson M, Wood S, Booth A, Rick J, Balain S (2007). A conceptual framework for implementation fidelity. Implement Sci.

[16] Sampling Manual for Facility Surveys for Population, Maternal Health, Child Health and STD Programs in Developing Countries. MEASURE Evaluation Manual Series, No. 3. MEASURE Evaluation. Carolina Population Center, University of North Carolina at Chapel Hill. 2001.

[17] Save the children. Standardized indicators and categories for better CMAM reporting 2015. Available at: https://docplayer.net/26996680-Standardised-indicators-and-categories-for-better-cmam-reporting.html.

[18] Tadesse E. Integrated community-based management of severe acute child malnutrition: Studies from rural Southern Ethiopia. Acta Universitatis Upsaliensis. 2016.

[19] Puett C, Guerrero S (2015). Barriers to access for severe acute malnutrition treatment services in Pakistan and Ethiopia: a comparative qualitative analysis. Public Health Nutr.

[20] Alvarez Moran JL, Alé FG, Rogers E, Guerrero S (2018). Quality of care for treatment of uncomplicated severe acute malnutrition delivered by community health workers in a rural area of Mali. Matern Child Nutr.

[21] Ali E, Zachariah R, Dahmane A, Van den Boogaard W, Shams Z, Akter T, Alders P, Manzi M, Allaouna M, Draguez B (2013). Peanut-based ready-to-use therapeutic food: acceptability among malnourished children and community workers in Bangladesh. Public health action.

[22] Appoh LY, Krekling S (2005). Maternal nutritional knowledge and child nutritional status in the Volta region of Ghana. Matern Child Nutr.

